# Towards a global strategy on digital health

**DOI:** 10.2471/BLT.20.253955

**Published:** 2020-04-01

**Authors:** Bernardo Mariano

**Affiliations:** a Digital Health and Innovation, World Health Organization, avenue Appia 20, 1211 Geneva 27, Switzerland.

The promise, opportunity and challenges of artificial intelligence in the health-care sector have captured the attention of academia, governments, civil society, the United Nations and nongovernmental organizations. The private sector, and more specifically the technology industry, is investing in artificial intelligence with acquisitions of life science companies and hiring health professionals to accelerate innovations in this field.^1^ Academia is introducing medical digital technologies in curricula and engaging in research programmes on digital health. Governments and nongovernmental organizations are actively engaged in many promising pilots, but are unable to align all pilots to countries’ needs and priorities.^2^

The tools, methods and technology used for big data and artificial intelligence are being applied to health services and systems globally.^3^ However, ethics and privacy issues dominate concerns regarding artificial intelligence.^4^ While these new technologies hold promise, this rapidly developing field raises transnational ethical, legal and social concerns about equitable access, privacy, appropriate uses and users, liability, bias and inclusiveness.^5^ Machine learning algorithms also pose novel ethical challenges for legislators and innovators who struggle to find the right balance for a trusted digital health ecosystem. Many questions remain about the ethical development and use of these technologies, including whether and how low- and middle-income countries will benefit from them. Although some government agencies,^6^ academic institutions,^7^^,^^8^ nongovernmental organizations^9^ and national ethics committees^10^ are addressing the ethical issues associated with the use of digital technology in health care, no international global guidance on ethics and artificial intelligence specific to health care exists.

Those privileging innovation and early adoption of these technologies are promoting the narrative of benefits outweighing risks. The risk of such arguments should not be underestimated, especially in low-resource settings, where regulatory measures are lagging as well as technical and governance capability. The role of a normative agency, such as the World Health Organization (WHO), as well as standardization agencies, such as the International Telecommunication Union and other such bodies, will be crucial to determine how the digital transformation of the health-care sector will improve quality of care, reduce health-care cost and increase accessibility to essential health-care services, in line with the universal health coverage (UHC) goals.

In May 2018, all WHO Member States passed a resolution on digital health.^11^ In response, WHO’s global digital health strategy sets a concrete framework for action to encourage international collaboration and regulation in the digital health ecosystem to achieve health for all.^12^

The four strategic objectives of the global strategy are to promote global collaboration and advance the transfer of knowledge on digital health; advance the implementation of national digital health strategies; strengthen governance for digital health at the global, regional and national levels; and advocate people-centred health systems that are enabled by digital health.

The strategy proposes a framework for regulating, benchmarking and certifying artificial intelligence and digital health medical devices, the same way WHO pre-qualifies medicines and vaccines, which involves full prequalification assessment of safety and efficiency, including quality control, testing, certification and reassessment to ensure consistent standards.

The strategy also calls for international health data regulations^12^ that consolidate health data as a global public health good, and outline principles of equitable data sharing for research and artificial intelligence to be the foundation of a safe, sustainable and innovative ecosystem, protecting the patients’ rights and improving health outcomes.

While WHO continues to work on its global strategy on digital health, several partnerships and groups are discussing digital health at an international level. Although most of these groups attempt to avoid duplication, it is inevitably taking place. The representation of all stakeholders in discussions of the opportunities, benefits, challenges and risks of digital health needs to be improved. 

The success of the digital transformation of the health-care sector will require all stakeholders to think of new ways of regulating, building trust and collaborating to leverage the potential of digital technologies to contribute to achieving national and global health goals. Success will depend on a multisectoral approach that includes all stakeholders, and on addressing their needs, and at times conflicting, objectives. The success of digital transformation in achieving UHC and the sustainable development goals will require all partners of the health ecosystem to find common grounds for collaboration in a safe and ethical digital ecosystem, guided by up-to-date principles of accessibility, affordability and quality health services. 
